# Single-Cell Reconstruction of Oxytocinergic Neurons Reveals Separate Hypophysiotropic and Encephalotropic Subtypes in Larval Zebrafish

**DOI:** 10.1523/ENEURO.0278-16.2016

**Published:** 2017-02-08

**Authors:** Ulrich Herget, Jose Arturo Gutierrez-Triana, Oriana Salazar Thula, Boris Knerr, Soojin Ryu

**Affiliations:** 1 Developmental Genetics of the Nervous System, Max Planck Institute for Medical Research, 69120 Heidelberg, Germany; 2 Focus Program Translational Neuroscience, University Medical Center, Johannes Gutenberg University Mainz, Langenbeckstr. 1, 55131, Mainz, Germany

**Keywords:** Brainbow, morphology, neuronal subtypes, oxytocin, zebrafish

## Abstract

Oxytocin regulates a diverse set of processes including stress, analgesia, metabolism, and social behavior. How such diverse functions are mediated by a single hormonal system is not well understood. Different functions of oxytocin could be mediated by distinct cell groups, yet it is currently unknown whether different oxytocinergic cell types exist that specifically mediate peripheral neuroendocrine or various central neuromodulatory processes via dedicated pathways. Using the Brainbow technique to map the morphology and projections of individual oxytocinergic cells in the larval zebrafish brain, we report here the existence of two main types of oxytocinergic cells: those that innervate the pituitary and those that innervate diverse brain regions. Similar to the situation in the adult rat and the adult midshipman, but in contrast to the situation in the adult trout, these two cell types are mutually exclusive and can be distinguished based on morphological and anatomical criteria. Further, our results reveal that complex oxytocinergic innervation patterns are already established in the larval zebrafish brain.

## Visual Abstract

**Figure:**
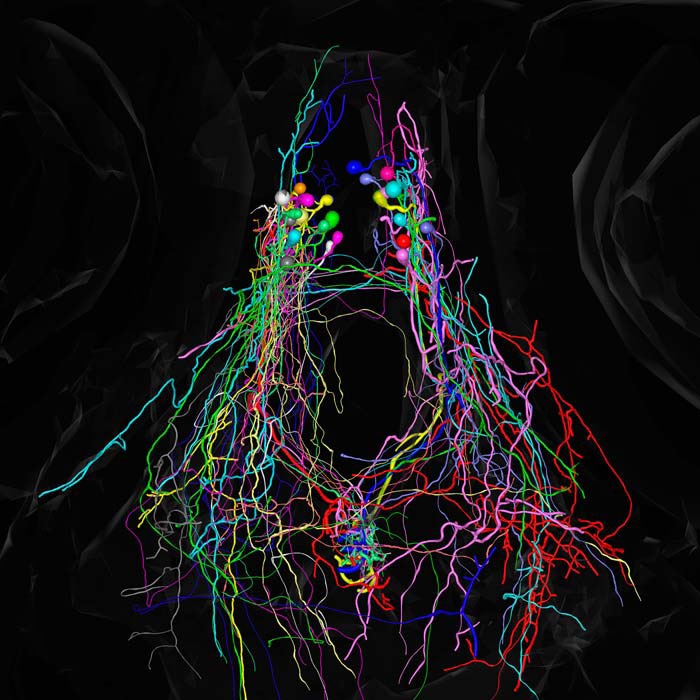

## Significance Statement

We used the Brainbow technique to reconstruct the morphology and projection patterns of oxytocinergic neurons in larval zebrafish, revealing diverse and complex brainwide innervation patterns originating from a small cluster of cells within the neurosecretory preoptic area. Central target areas include the tectum, hypothalamus, and telencephalon. This is the first comprehensive whole-brain morphology characterization of oxytocinergic neurons, to our knowledge. 3D registration reveals spatially distinct subtypes of oxytocinergic neurons. One group with morphologically complex projections reaches into distinct brain regions, presumably for neuromodulation, whereas another group features simpler projections innervating the pituitary, presumably for endocrine release. These two groups are spatially segregated, suggesting an evolutionarily ancient anatomical separation of oxytocin cell subtypes.

## Introduction

Oxytocin (Oxt; formerly isotocin) is exclusively produced by a small group of neurons in the zebrafish preoptic hypothalamus (Herget et al., 2014), yet it regulates diverse evolutionarily conserved functions. Neurons producing Oxt in the rat are activated during stress or feeding, and Oxt is involved in analgesia and energy metabolism (Lee et al., 2009; Onaka et al., 2012; Eliava et al., 2016). Oxt’s most prominent role is the regulation of social behavior and reproduction, including conspecific recognition, pair bonding, fear and trust regulation, mother–child attachment, parturition, and lactation in mammals (Pedersen and Prange, 1979; Kendrick et al., 1987; Lee et al., 2009; Onaka et al., 2012). Its prominent social regulatory role is further supported by its potential involvement in the impairment of social interaction in autism spectrum disorders in humans (Meyer-Lindenberg et al., 2011).

Oxt is produced in the paraventricular (PVN) and supraoptic nuclei of the mammalian hypothalamus and transported through axons toward the neurohypophysis, the neuronal posterior lobe of the pituitary, where it is released into the body circulation (Brownstein et al., 1980). Oxt is also transported and released within the brain, probably via two different delivery modes: local dendritic release followed by slow diffusion (Ludwig and Leng, 2006) and fast targeted axonal release in distant regions widely spread throughout the brain (Buijs, 1978; Sofroniew, 1980; Swanson and Hartman, 1980; Knobloch et al., 2012). For example, Oxt originating from rat hypothalamic cells is axonally delivered to forebrain regions including the central amygdala, where Oxt mediates fear suppression (Knobloch et al., 2012). Such Oxt-transporting projections have also been suggested in voles and mice (Ross et al., 2009; Dölen et al, 2013). Recent observations in rats also suggest mutual innervation of oxytocinergic cells within and across hemispheres (Eliava et al., 2016).

Despite these advances, it is not well understood how central and peripheral functions of Oxt are coordinated. Some studies demonstrated that magnocellular Oxt neurons of the neurohypophyseal system feature collaterals reaching into the striatum or central amygdala (Ross et al., 2009; Knobloch et al., 2012). Some oxytocinergic cells were recently demonstrated to have a dual analgesic function in rats, innervating other neuroendocrine oxytocinergic cells to release Oxt into the blood, while simultaneously inhibiting nociception by long-range collateral fibers reaching into the dorsal horn of the lumbar spinal cord (Eliava et al., 2016). Although a dichotomic separation of PVN projections reaching toward either the pituitary or the medulla and spinal cord was suggested before (Hosoya and Matsushita, 1979; Swanson and Kuypers, 1980), conflicting data were reported in fish (separate projection cell types in the plainfin midshipman, Goodson et al., 2003; dual projections of the same cells in the trout, Saito et al., 2004). The situation in the developing zebrafish brain is unknown. An important and currently open question is whether distinct Oxt neuron subtypes exist in zebrafish that mediate specific central or peripheral functions. To address this question, a large number of Oxt-positive cells must be sampled, and individual Oxt cells must be categorized based on distinguishable features.

With a simpler transparent brain and genetic access, zebrafish larvae offer significant experimental advantages for structural and functional analysis of individual neurons. Importantly, zebrafish larvae execute endocrine functions and complex behaviors within their first 5 d of development (Löhr and Hammerschmidt, 2011). Although the presence of tracts and projections throughout brain regions caudal to the PVN-homologous neurosecretory preoptic area (NPO) was shown before (Coffey et al., 2013; Herget et al., 2014), the morphology and target regions of individual oxytocinergic neurons have remained unclear.

In this study, we used zebrafish larvae to characterize the morphology and projection targets of neurons producing Oxt. To distinguish individual cells within the dense cluster and fiber bundles, we targeted Brainbow (Livet et al., 2007; Lichtman et al., 2008) to the NPO (Herget et al., 2014) of 6-d-old zebrafish larvae using both an *oxt* promoter (Coffey et al., 2013) and a conserved regulatory element of the transcription factor *orthopedia a* (*otpa*; Gutierrez-Triana et al., 2014). Random recombination of the Brainbow cassette by inducible Cre recombinase allowed spectral separation of individual cells and their projections from their densely intermingled neighbors. Morphological whole-cell 3D skeletonization revealed a striking structural diversity and multiple target regions of Oxt-producing neurons in the larval zebrafish. Registration of skeletonized cells into one common model brain showed a separation of the oxytocinergic cluster into two distinct groups: a rostral hypophysiotropic cluster and a caudal encephalotropic cluster, indicating that in zebrafish, peripheral neuroendocrine and different central neuromodulatory processes are likely mediated by dedicated oxytocinergic pathways.

## Methods

### Fish maintenance

Maintenance and breeding of male and female AB/TL wild-type zebrafish were performed under standard conditions at 28.5°C on a 12:12-h light/dark cycle (Westerfield, 2000). For all experiments, crosses of AB and TL strains were used. All procedures were performed according to the guidelines of the German animal welfare law and approved by the local government (Regional Council, Karlsruhe, Germany).

### Plasmid construction

The pST_OtpECR6:BrbM plasmid was generated by cloning the 3× multimerized NPO-regulatory region of the zebrafish gene *otpa* (*OtpECR6*) together with the *E1B* minimal promoter (Gutierrez-Triana et al., 2014) upstream of the membrane-bound Brainbow-1.1 (BrbM) cassette (Livet et al., 2007) in a vector containing both I-SceI and Tol2 sites. To generate pST_Oxt*:*BrbM, the *OtpECR6* enhancer was replaced by the 1.7-kb regulatory region of the zebrafish *oxt* gene, whose activity was shown to reflect the expression of endogenous *oxt* (Coffey et al., 2013).

### Generation of transgenic lines

Recombinant plasmids were injected into one-cell-stage AB/TL embryos at a 15 ng/μl concentration in the presence of 10 ng/μl Tol2 transposase mRNA and 0.05% phenol red (Sigma-Aldrich, #P3532). The progenies of injected fish were maintained in E2 medium + 0.2 mm *N*-phenylthiourea (Sigma-Aldrich, #P7629) to prevent pigmentation and screened for preoptic Kusabira expression at 3 d post-fertilization (dpf) using a Leica MZ6 fluorescence microscope. The following stable transgenic lines were established: *Tg(OtpECR6:BrbM)^hd23^* and *Tg(Oxt:BrbM)^hd24^.* Both transgenic lines were incrossed for multiple generations to increase copy numbers.

### Brainbow induction

For stochastic recombination of the Brainbow transgenes, one-cell-stage progenies of *Tg(OtpECR6:BrbM)^hd23^* or *Tg(Oxt:BrbM)^hd24^* fish were injected with hsp:nls-Cre plasmids at a concentration of 15 ng/μl in the presence of 10 ng/μl I-SceI meganuclease enzyme and 0.05% phenol red. For transient Brainbow expression, we injected one-cell-stage AB/TL embryos with pST_OtpECR6:BrbM or pST_Oxt:BrbM plasmids at a concentration of 15 ng/μl together with 10 ng/μl Tol2 transposase mRNA and hsp:nls-Cre plasmid with I-SceI enzyme as described above. On the next day, injected embryos were heat-shocked at 37°C for 1.5–15 h, depending on the line used and the sparseness desired.

### Whole-mount immunohistochemistry

Fixation and staining were performed as reported previously (Kastenhuber et al., 2010; Herget et al., 2014), using a chicken anti-GFP antibody (Abcam, #13970, RRID: AB_300798) together with a rabbit anti-Oxt antibody (Herget et al., 2014), and anti-chicken Alexa Fluor 488 (Invitrogen, #11039, RRID: AB_142924) plus anti-rabbit Alexa Fluor 647 secondary antibodies (Invitrogen, #A21245, RRID: AB_141775). Anatomical reference landmarks were visualized using NeuroTrace Fluorescent Nissl Stain (NT; Invitrogen, #N21480).

### Confocal microscopy

For imaging, larval heads were cleared in 80% glycerol (Gerbu Biotechnik, #2006.50000) in PBS for 1 h. Confocal stacks were recorded using a Leica SP5 confocal microscope with a 20× glycerol objective. Live imaging was performed on agarose-embedded larvae (Low Melt Agarose, Carl Roth, #6351.1) with a 20× water objective. Each channel was recorded sequentially to reduce interfering signals from overlapping emission spectra. Zoom, dimensions, gain, offset, average, and speed were adjusted for each stack to obtain the optimal image quality of the desired volume.

### Image evaluation

Stacks were evaluated using Amira 5.3 or 5.4 (FEI Visualization Sciences Group) to create maximum intensity projections and 3D voxel-rendered views and to perform 3D registration and neuron skeletonization. Maximum intensity projections and voxel-rendered views were restricted to the volume of interest. Brightness and contrast were adjusted for each channel. Neuron skeletonization was performed using the SkeletonTree plugin (Schmitt et al., 2004; Evers et al., 2005). Stacks of different animals were manually registered by transformation using commonly stained cell clusters as references.

## Results

### Complex oxytocinergic projections are present during early development in larval zebrafish

**Figure 1.**
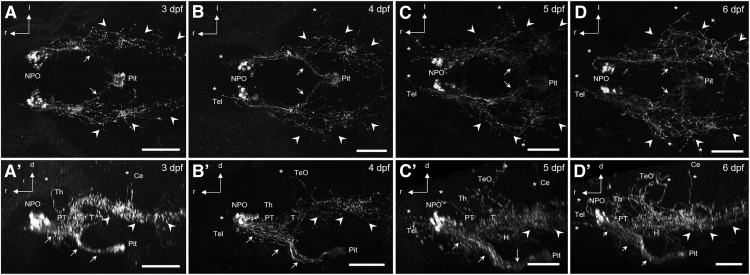
Projections of Oxt-positive cells in the NPO of developing zebrafish. Dorsal (***A–D***) and lateral (***A′–D′***) views of immunostained Oxt-positive cells and their projections. Note the complexity of projections reaching toward the pituitary (arrows), the spinal cord (arrowheads), and other brain regions (asterisks). Pituitary and spinal projections are established at 3 dpf (*n* = 9), whereas additional innervation of other brain regions forms over 4 (*n* = 11) and 5 (*n* = 13) dpf and is established by 6 dpf (*n* = 24). Scale bars: 100 μm. d, dorsal (following body axis); H, hypothalamus; l, lateral; Pit, pituitary; PT, posterior tuberculum; r, rostral (following body axis); T, tegmentum; Th, thalamus.

**Figure 2.**
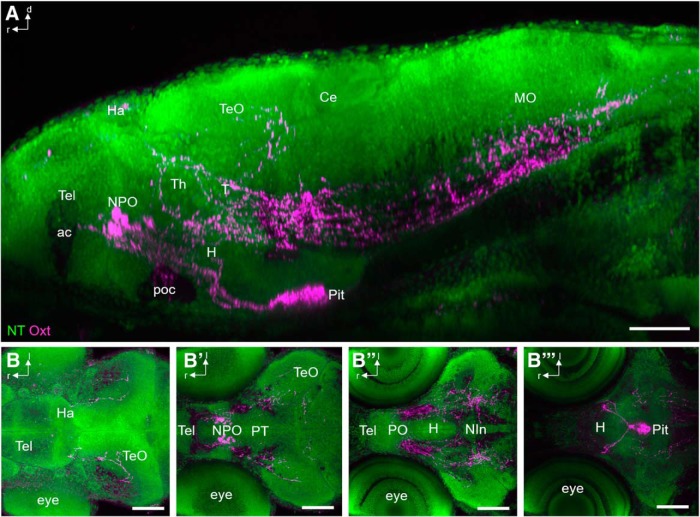
Innervated regions of Oxt-positive neurons at 6 dpf. ***A***, Lateral view of immunostained Oxt-carrying fibers (magenta) in combination with NT (green) to identify brain regions at 6 dpf (*n* = 12). Fibers reach the pituitary through the hypothalamohypophyseal tract, but also innervate the caudal Tel and TeO. Spinal projections pass the metencephalon and myelencephalon ventrally. ***B–B″′***, Serial horizontal substacks (dorsal to ventral) show innervation of the TeO (B), the caudal Tel (***B′***), hypothalamic and tegmental regions (***B″***), and the pituitary (***B″′***). The fibers mostly traverse within Nissl-negative regions, which correspond to white matter. Scale bars: 100 µm. ac, anterior commissure; Ha, habenula; MO, medulla oblongata; NIn, interpeduncular nucleus; poc, postoptic commissure.

**Figure 3.**
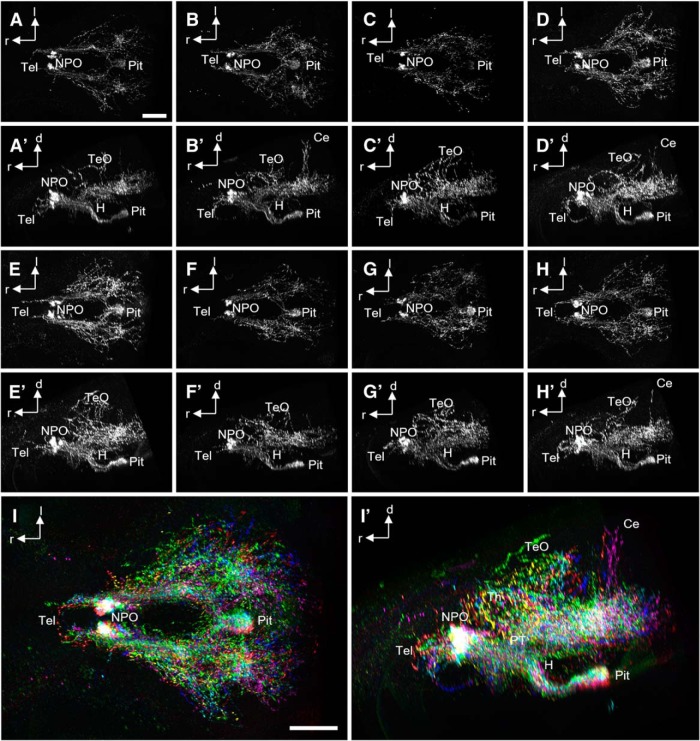
Variable Oxt-positive projections at 6 dpf. ***A–H′***, Oxytocinergic cells and their innervation patterns shown by IHC in eight different animals, dorsal (***A–H***) and lateral (***A′–H′***) views. ***I***, ***I′***, The same data after 3D registration of all stacks into one common model, dorsal (***I***) and lateral (***I′***) views. Each stack is shown with a different color. Note the variability of innervation patterns. Some animals show oxytocinergic innervation of the Ce, and tectal branches appear to be somewhat different in each individual. Scale bars: 100 µm.

Because of the successive specialization of Oxt functions during evolution, ascending Oxt projections are thought to be a feature of more advanced and mature vertebrates (Knobloch and Grinevich, 2014). To determine the extent of Oxt projection complexity in developing larvae, we used a custom-made Oxt antibody (Herget et al., 2014) to characterize the development of Oxt projection patterns in larvae at 3–6 dpf. In larval zebrafish, oxytocinergic somata are restricted to the NPO (Fig. 1 and Herget et al., 2014). Their projections form densely entangled bundles innervating the pituitary, but also reach into other brain regions and toward the spinal cord (Fig. 1). Strikingly, distinct fibers can be observed reaching into the optic tectum (TeO), the hypothalamus, and the telencephalon (Tel) at these early developmental stages. Hypophyseal and spinal projections can be observed from 3 dpf on (Fig. 1*A*, *A′* ), whereas other brain regions are innervated beginning at 4–5 dpf (Fig. 1*B–C′*). The innervation patterns observed by 6 dpf (Fig. 1*D*, *D′* ) remain stable until at least 8 dpf (data not shown). Target regions were identified using NT as a cytoarchitectural marker of brain anatomy (Fig. 2). In 6 dpf larvae, oxytocinergic fibers were found reaching into the Tel close to the anterior commissure (Fig. 2*A*, *B′*), crossing through the postoptic commissure (Fig. 2*A*), prominently innervating the pituitary via the hypothalamohypophyseal tract (Fig. 2*A*, *B″′* ), passing through major parts of the thalamus, tegmentum, and hypothalamus (Fig. 2*A*, *B′*, *B″* ), reaching dorsally up into the TeO (Fig. 2*A–B′*), or passing ventrally through the medulla oblongata (Fig. 2*A*). Dense collateral branching occurs in the medulla oblongata, whereas only few unbranched fibers appear to run laterally parallel to the spinal cord, and therefore potentially within the putative sympathetic trunk (data not shown). Generally, Oxt immunostaining showed regional interindividual variability at this developmental stage, especially for the branches reaching into the TeO and the cerebellum (Ce; Fig. 3).

### The Brainbow technique allows morphological reconstruction of individual oxytocinergic neurons and their fibers

**Figure 4.**
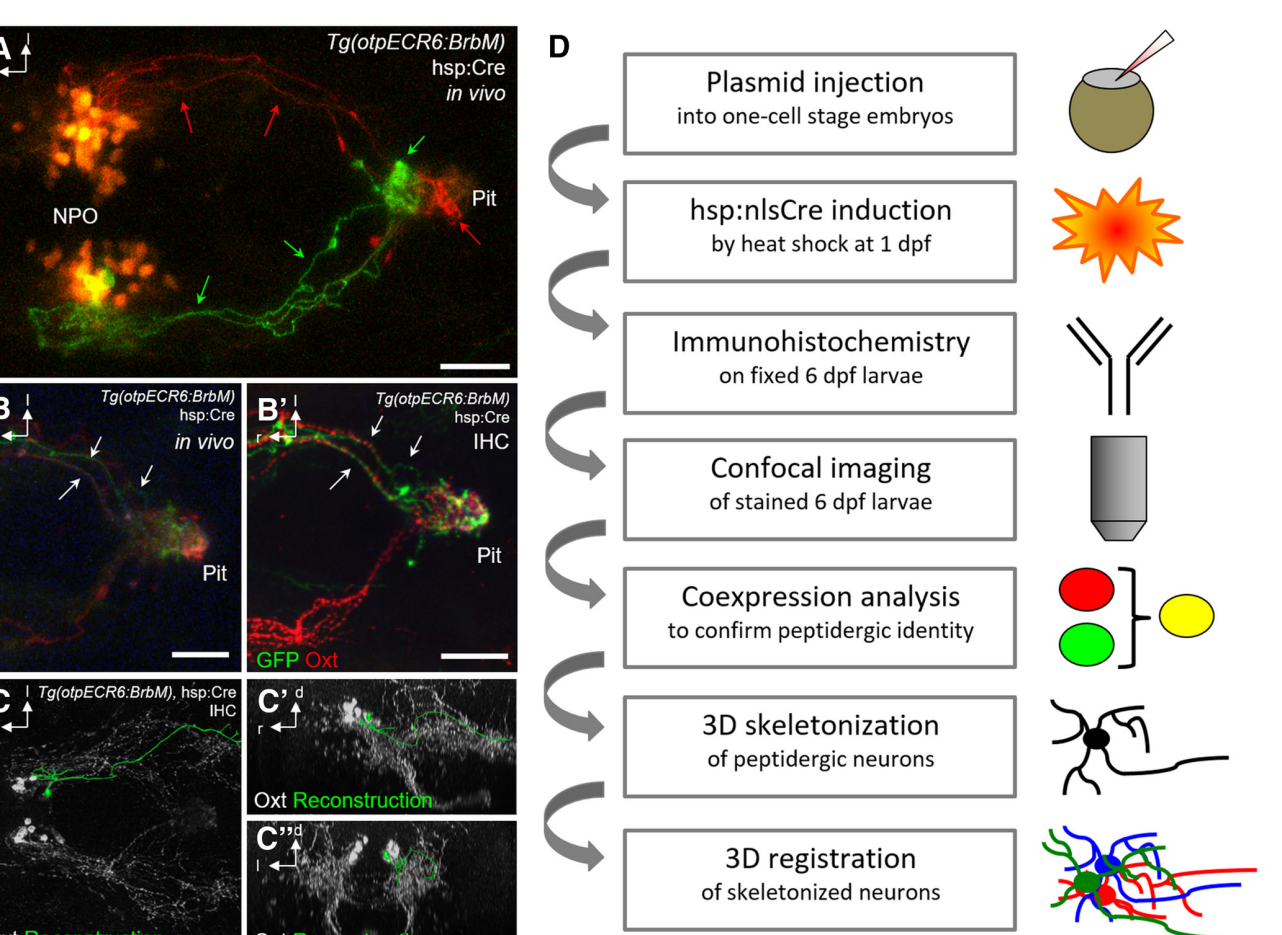
NPO Brainbow in larval zebrafish. ***A***, Different colors resulting from recombination distinguish different projections and target regions (arrows) of NPO cells towards the pituitary at 5 dpf, which would be unrecognizable with only one color. Scale bars: 50 μm. ***B–B′***, Innervation of the pituitary shown in *Tg(otpECR6:BrbM)* at 4 dpf *in vivo* (***B***), and after IHC staining for GFP and Oxt (***B′***). Note that features of the projections visible *in vivo* can also be identified after fixation and staining (arrows). ***C–C″***, Reconstructed morphology of an Oxt-positive cell resulting from Brainbow recombination (green), shown together with Oxt IHC (white) in dorsal (***C***), lateral (***C′***), and frontal views (***C″***). This cell projects toward the spinal cord. ***D***, Methodological pipeline. One-cell-stage embryos (*n* > 5,000) were injected and subjected to a heat shock on the next day to induce Cre recombinase. The surviving embryos (∼3,500) were fixed at the relevant stage and immunostained to confirm peptidergic cell type identity. Brainbow recombination was displayed in 181 animals, and recombination occurred in oxytocinergic cells in 119 animals, covering a total of 216 Oxt-positive cells. This subset of animals showing successful recombination in oxytocinergic cells was used for single-cell skeletonization. Cells that could not be completely reconstructed with certainty were discarded, so eventually 26 animals were used for the reconstructions presented. Reconstructed cells were registered using common landmarks. Note that each selection step excludes animals from further processing, requiring a large starting number to get few cell reconstructions and resulting in overall low efficiency. Scale bars: 50 μm.

An immediate question raised by the complex arrangement of oxytocinergic fiber bundles is which soma connects to which brain region. This dense network of fibers can be dissected only by single-cell reconstruction. To visualize individual Oxt neurons in their entirety, we used the Brainbow technique to achieve multicolor labeling of the entire oxytocinergic neuron population. We used two independent transgenic approaches. We began by using Brainbow driven by a promoter covering the NPO (Gutierrez-Triana et al., 2014), and later also used an Oxt promoter which restricts the Brainbow expression to mostly oxytocinergic cells (Coffey et al., 2013). We achieved Brainbow expression in the NPO (Fig. 4*A*) by linking BrbM to the *otpECR6* promoter, a conserved regulatory element of the transcription factor *otpa*, which allows genetic targeting of a part of the NPO covering *corticotropin-releasing hormone* (*crh*), *arginine vasopressin* (*avp*), and *oxt*-positive cells (Gutierrez-Triana et al., 2014). The established Brainbow line, *Tg(OtpECR6:BrbM)*, can be used to distinguish the innervation targets of different NPO cells, as illustrated by differently colored cells projecting to different neurohypophyseal subregions (Fig. 4*A*). Because Brainbow is expressed throughout the NPO and therefore covers neighboring and intermingled cell types as well, immunohistochemistry (IHC) was carried out using the Oxt antibody together with an anti-GFP antibody (to detect recombined BrbM fluorescent proteins) to confirm oxytocinergic identity. Comparing confocal images taken *in vivo* with images of the same animal after fixation and staining reveals fibers carrying the peptide labeled by IHC (Fig. 4*B*, *B′* ). IHC also greatly improves the fluorescence signal and reduces the background, facilitating visualization of fibers in deep regions such as the pituitary. In addition to *Tg(OtpECR6:BrbM)*, we generated *Tg(Oxt:BrbM)* using a published *oxt* promoter (Coffey et al., 2013), which is more specific for oxytocinergic cells. IHC confirmation of oxytocinergic identity was nevertheless also performed in experiments using this *oxt* promoter to ensure that only oxytocinergic cells were processed further for reconstruction. We also used animals that transiently expressed Brainbow (for higher expression levels at the expense of color diversity). The morphology of nonoverlapping cells identified as Oxt-positive by IHC was reconstructed using Amira (Fig. 4*C–C″*). Several selection steps excluded animals from further processing (Fig. 4*D*). We often observed expression of the same fluorophore in neighboring cells or induction of Brainbow in fibers not carrying Oxt, and such animals were excluded from morphological analysis. Only those animals displaying successful recombination in cells positive for Oxt that were separated from other costained cells allowed the reconstruction of individual oxytocinergic neurons and their projections. Cases in which a cell was partially but not completely reconstructable were excluded from further morphological analysis but included in soma position analysis.

### Morphological analysis of oxytocinergic cells reveals two major types based on their projection patterns

**Figure 5.**
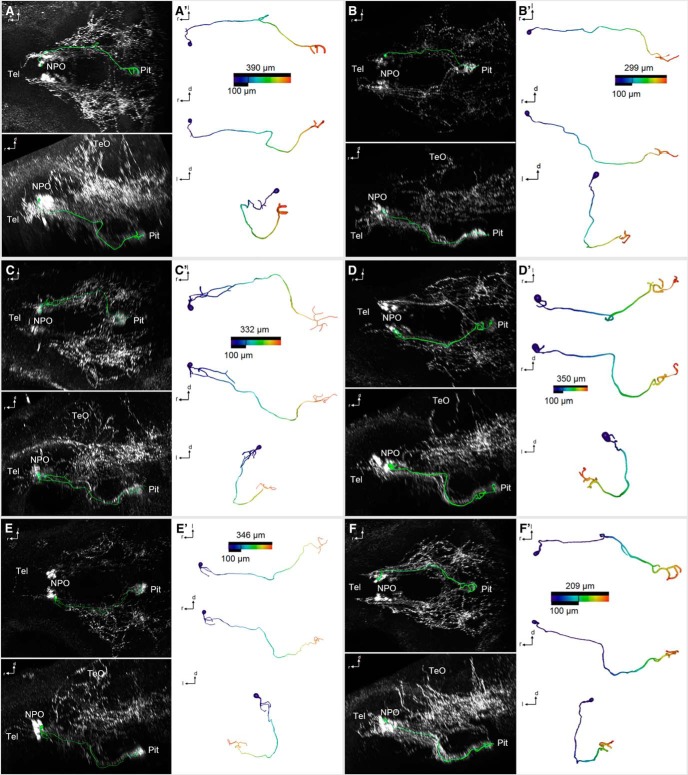
3D reconstruction of morphologically simple Oxt-positive preoptic neurons that innervate the pituitary at 6 dpf. ***A–F″***, Skeletonized neurons, shown within the context of the Oxt IHC (***A–F***; dorsal and lateral views), and shown individually with a color code of the distance along each branch from the soma center (***A′–F′***; dorsal, lateral, and frontal views). Distance color codes are scaled to the maximum distance of each cell. Note that these cells feature very limited branching close to the soma, or no branching at all, until they reach the pituitary.

**Figure 6.**
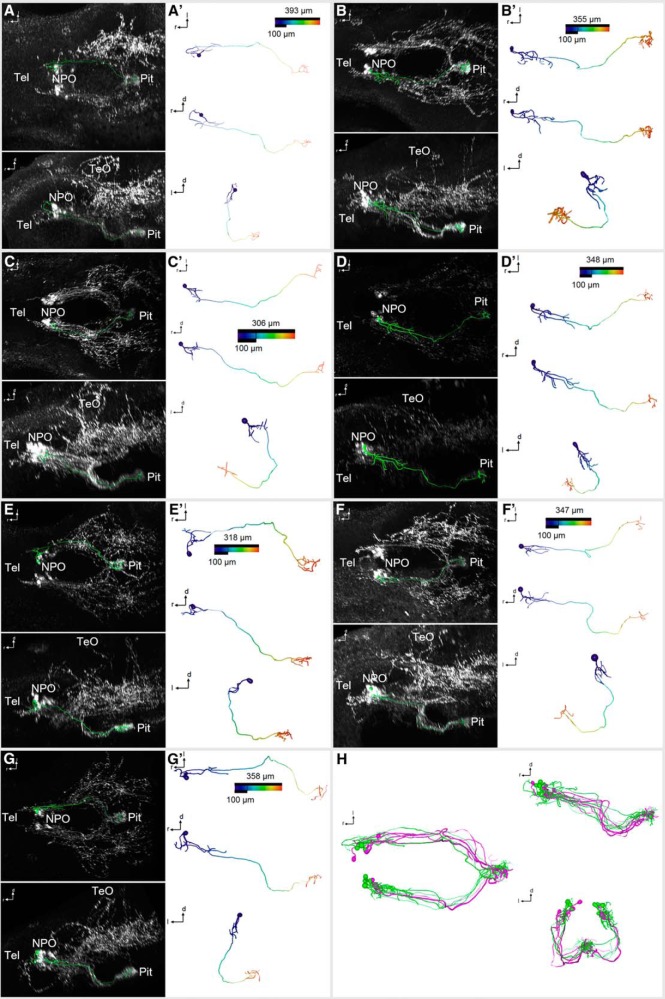
3D reconstruction of morphologically complex Oxt-positive preoptic neurons that innervate the pituitary at 6 dpf. ***A–G″***, Skeletonized neurons, shown within the context of the Oxt IHC (***A–G***; dorsal and lateral views), and shown individually with a color code of the distance along each branch from the soma center (***A′–G′***; dorsal, lateral, and frontal views). Distance color codes are scaled to the maximum distance of each cell. Note that these cells feature several branches close to the soma. ***H***, Registration of the hypophysiotropic cells shown in Fig. 5 (magenta) and Fig. 6 (green) shows intermingled projections (dorsal, lateral, and frontal views).

Using the Brainbow approach, we characterized the morphological diversity and projection targets of 6-dpf oxytocinergic cells and found diverse projection patterns that can be categorized into two distinct groups: some neurons innervate the pituitary, and others instead innervate other brain regions. Oxt-positive cells innervating the pituitary are comparatively simple, featuring only a single projection toward the pituitary, or very limited branching near the soma within the NPO (Fig. 5), which occasionally extends caudally (Fig. 5*C*, *C′* ; putative posterior tuberculum or rostral hypothalamus). Other hypophysiotropic cells feature more complex proximal branching within the NPO (Fig. 6*A–G′*), which often extends caudally (Fig. 6*B*, *B′*, *D*, *D′*, *F*, *F′*, *G*, *G′* ; putative posterior tuberculum or rostral hypothalamus). The somata and projections of these two subcategories do not segregate after registration (Fig. 6*H*; see also below).

**Figure 7.**
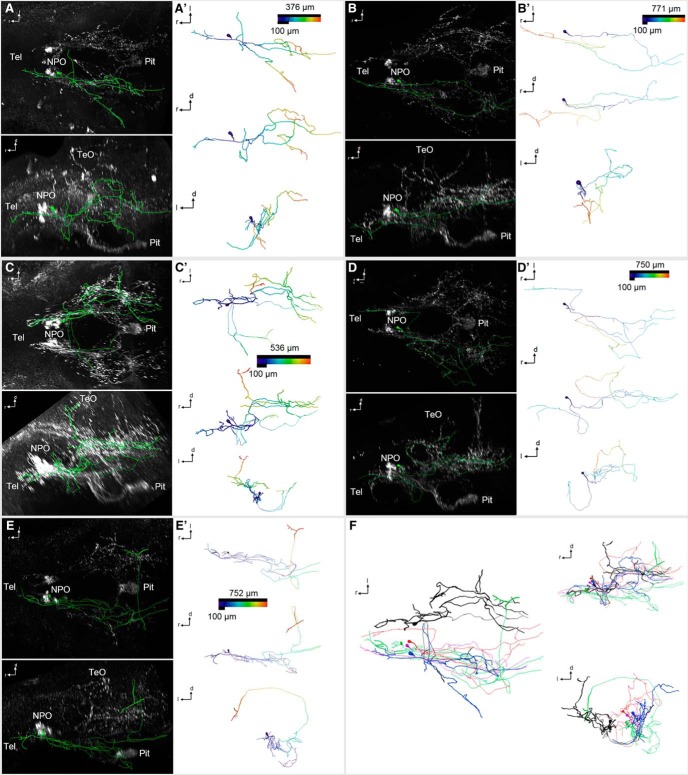
3D reconstruction of Oxt-positive preoptic neurons that innervate the Tel and other brain regions, but not the pituitary, at 6 dpf. ***A–E″***, Skeletonized neurons, shown within the context of the Oxt IHC (***A–E***; dorsal and lateral views), and shown individually with a color code of the distance along each branch from the soma center (***A′–E′***; dorsal, lateral, and frontal views). Distance color codes are scaled to the maximum distance of each cell. Note that these cells feature extensive branching throughout the brain, with fibers reaching rostrally into the Tel (***A–E′***), caudoventrally into the hypothalamus (***A–C′***, ***E–E′***), caudodorsally into the TeO (***C–D′***), and several of the branches crossing to the contralateral hemisphere (***A–A′***, ***C–E′***). ***F***, Registration of these encephalotropic cells shows the contribution of each cell to the brainwide innervation pattern (dorsal, lateral, and frontal views).

**Figure 8.**
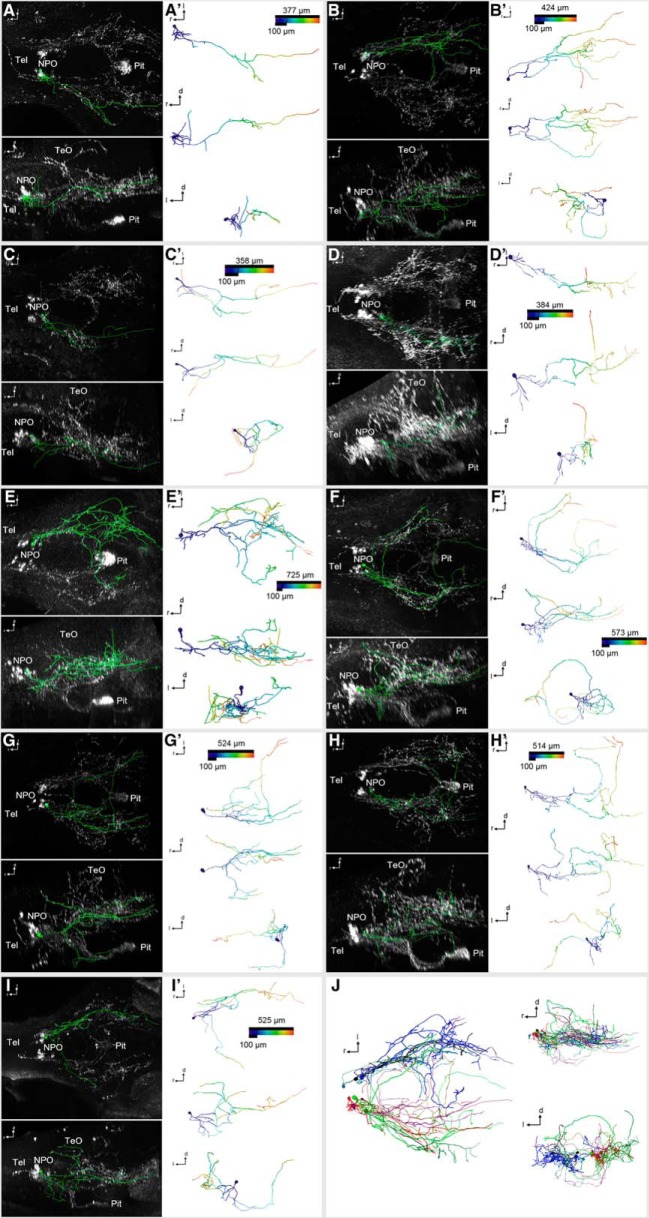
3D reconstruction of Oxt-positive preoptic neurons that innervate other brain regions at 6 dpf. ***A–I′***, Skeletonized neurons, shown within the context of the Oxt IHC (***A–I***; dorsal and lateral views), and shown individually with a color code of the distance along each branch from the soma center (***A′–I′***; dorsal, lateral, and frontal views). Distance color codes are scaled to the maximum distance of each cell. Note that these cells feature extensive branching throughout the brain, with fibers reaching dorsally into the prethalamus (***A***, ***A′***), caudoventrally into the hypothalamus (***B–I′***), caudodorsally into the Ce (***D***, ***D′***) and the TeO (***G–I′***), and several of the branches crossing to the contralateral hemisphere (***B***, ***B′***, ***E–I′***). ***J***, Registration of these encephalotropic cells shows the contribution of each cell to the brainwide innervation pattern (dorsal, lateral, and frontal views).

Apart from pituitary-innervating cells, broad branching throughout the brain was observed for other Oxt-positive neurons (Figs. 7 and 8). Projections of some cells reached rostrally into the Tel and other brain regions, but not the pituitary (Fig. 7). These cells feature extensive branching throughout the brain, with fibers reaching rostrally into the Tel (Fig. 7*A–E′* ), caudoventrally into the hypothalamus (Fig. 7*A–C′*, *E–E′*), caudodorsally into the TeO (Fig. 7*C–D′*), and branches crossing to the contralateral hemisphere (Fig. 7*A*, *A′*, *C–E′* ; registration in *F*). Other encephalotropic cells did not reach into the Tel or toward the pituitary, but into other brain regions (Fig. 8). These cells featured projections reaching dorsally into the prethalamus (Fig. 8*A*, *A′* ), caudoventrally into the hypothalamus (Fig. 8*B–I′* ), caudodorsally into the Ce (Fig. 8*D*, *D′* ) and the TeO (Fig. 8*G–I′* ), and branches crossing to the contralateral hemisphere (Fig. 8*B*, *B′*, *E–I* ; registration in *J*). Strikingly, we never observed Oxt cells that projected to both the brain and the pituitary. Therefore, these two projection types are mutually exclusive and occur in different cells.

### Neuroendocrine and neuromodulatory clusters exhibit anatomical separation

**Figure 9.**
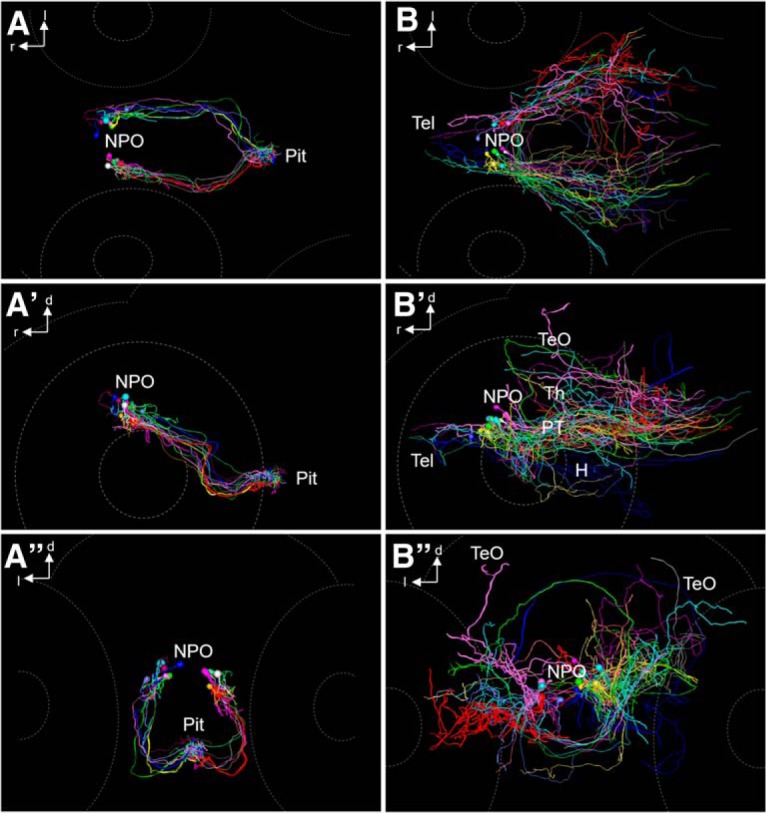
Projections of Oxt-positive cells manually registered from different 6 dpf animals into one common model. ***A–A″***, Oxt-positive cell morphology innervating the pituitary, dorsal (***A***), lateral (***A′***), and frontal (***A″***) views. Each cell is shown with a different color. A 3D rotation of this dataset is provided as Movie 1. ***B–B″***, Oxt-positive cell morphology innervating regions other than the pituitary, dorsal (***B***), lateral (***B′***), and frontal (***B″***) views. Each cell is shown with a different color. A 3D rotation of this dataset is provided as Movie 2.

**Figure 10.**
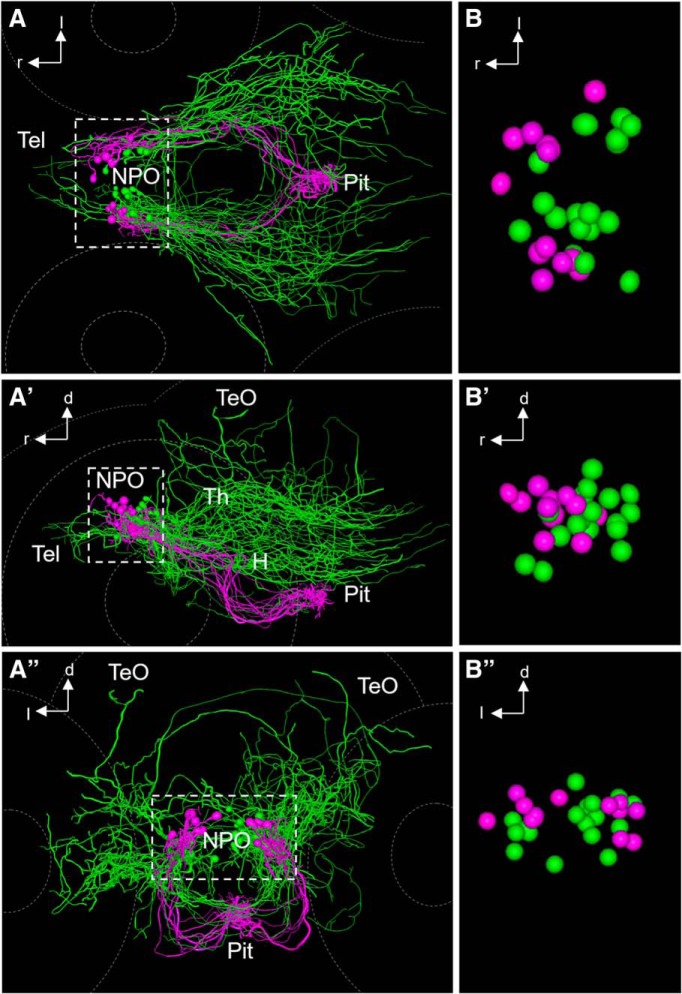
Comparison of hypophysiotropic and encephalotropic cell types after registration. ***A–A″***, Comparison of encephalotropic (green) and hypophysiotropic (magenta) innervation of Oxt-producing cells, dorsal (***A***), lateral (***A′***), and frontal (***A″***) views. A 3D rotation of this dataset is provided as Movie 3. B–B″, Magnified view of reconstructed soma centers after registration, dorsal (***B***), lateral (***B′***), and frontal (***B″***) views. Three additional somata of encephalotropic oxytocinergic cells were added, since the soma was reconstructable. Note that the two morphological subtypes appear to possess segregated somata, suggesting a functionally relevant regionalization within the cluster of Oxt-producing cells. Hypophysiotropic cells reside in a more rostral part of the NPO compared with encephalotropic cells.

To determine whether Oxt cells that exhibit the two projection types show anatomical clustering, we visualized the cell bodies and projection patterns of groups of cells. We manually registered reconstructed cells by 3D transformation using both the residual Kusabira signal of unrecombined Brainbow and the costained peptide IHC labeling in the NPO and the pituitary as reference landmarks. This allowed the visualization of multiple cells within the same model brain (Fig. 9; see also Figs. 6*H*, 7*F*, and 8*J*). The trajectory of axons innervating the pituitary generally follows a similar double-curved path, first turning ventrally but then curving dorsally and into the pituitary (Fig. 9*A–A″*; Movie 1). In comparison, the combined group of encephalotropic cells shows a much more diverse collection of morphologies, with each cell featuring a unique innervation pattern (Fig. 9*B–B″*; Movie 2). Comparing the hypophysiotropic and encephalotropic groups of all reconstructed cells after registration revealed that the hypophysiotropic population resides in a more rostral part of the NPO, whereas the encephalotropic cells tend to be caudal to them (Fig. 10; Movie 3). This suggests an anatomical separation of these subtypes of oxytocinergic cells within the NPO.

Movie 1. 3D rotation showing projections of hypophysiotropic Oxt-positive cells. Morphological reconstructions were manually registered from different 6 dpf animals into one common model. Each cell is shown with a different color. See also Fig. 9*A–A″*. 10.1523/ENEURO.0278-16.2016.video.1

Movie 2. 3D rotation showing projections of encephalotropic Oxt-positive cells. Morphological reconstructions were manually registered from different 6-dpf animals into one common model. Each cell is shown with a different color. See also Fig. 9*B–B″*. 10.1523/ENEURO.0278-16.2016.video.2

Movie 3. 3D rotation comparing hypophysiotropic and encephalotropic cell types. Morphological reconstructions were manually registered from different 6-dpf animals into one common model. The soma positions and projection patterns of hypophysiotropic cells (magenta) and encephalotropic cells (green) are separated. See also Fig. 10*A–A″*. 10.1523/ENEURO.0278-16.2016.video.3

## Discussion

Oxt is thought to be delivered via different release pathways: volume transmission from hypothalamic dendrites (Ludwig and Leng, 2006) or targeted axonal long-range delivery. Our understanding of distinct innervation patterns and the circuitry of different Oxt functions has been rapidly increasing due to considerable recent advances using rats. Innervated brain regions were reported >30 years ago in rats (Buijs, 1978; Sofroniew, 1980; Swanson and Hartman, 1980), but functionally specific circuits have been identified only in recent years (Knobloch et al., 2012; Eliava et al., 2016).

The subgroup of oxytocinergic neurons featuring projections to distant brain regions is thought to be anatomically distinct from hypophysiotropic neurons (Hosoya and Matsushita, 1979; Swanson and Kuypers, 1980), leading to the assumption that in specific situations (e.g., empathy, fear, pain, stress), dedicated subpopulations of neurons would modulate relevant brain regions to guide behavior toward survival of the individual and the species (Knobloch and Grinevich, 2014). However, the direct demonstration of distinct Oxt cell types based on their projections has been lacking, since the morphological diversity of oxytocinergic cells underlying circuit function is very difficult to extract from the large and opaque brains of rodents. Here we provide anatomical and morphological evidence for the existence of two major types of Oxt cells with distinct projections in larval zebrafish.

To visualize individual neurons in their entirety, two methods are commonly used: sparse single-color labeling and multicolor labeling of a group of neurons using Brainbow. Sparse labeling has been used in zebrafish to define dopaminergic (Tay et al., 2011) and olfactory projectomes (Miyasaka et al., 2014), yet such an approach depends on transient expression. The Brainbow method is an elegant tool for morphological reconstruction of multiple cells in larval zebrafish (Robles et al., 2013), since it allows multicolor labeling in stable transgenic lines depending only on Cre recombinase, which can be provided via heat shock induction. In theory, this approach should allow more efficient single-cell reconstruction, but its actual efficiency in NPO cells was overall very low due to multiple selection steps that excluded many animals from further analysis (Fig. 4*D*). Because our results show that immunostaining is required for the confirmation of cell type identity and signal enhancement in deep regions, for future studies we suggest the use of separately detectable fluorophores for independent immunostaining, as reported previously (Cai et al., 2013). Although the registration approach we used might raise concerns about repeated sampling of the same cell type, we assume that the stochastic nature of random Brainbow recombination prevents such potential sampling bias. Oversampling of the same cell type is also unlikely because of considerable interindividual differences observed in cell numbers, soma positions, hemispheric distribution, innervation patterns, and coexpression levels across animals. The cells shown here are a small subset of a large dataset of imaged and reconstructed samples that included many unreconstructable or only partially reconstructable cells (because of overlap or crossing of branches with the same labeling). Nevertheless, in all these images and partial reconstructions, we saw the same two major types shown here.

Our results suggest that long-range projections of Oxt neurons are ancestral in the vertebrate brain, and that central Oxt projections are present during early development in zebrafish, unlike in developing mammals (Grinevich et al., 2015, 2016). Our data are partially consistent with an earlier study in the rainbow trout, in which oxytocinergic cells also project widely throughout the brain, innervating ventral telencephalic, diencephalic, and mesencephalic regions in addition to the pituitary (Saito et al., 2004). Each of the analyzed cells projected to both the pituitary and central brain regions, suggesting that peripheral endocrine and central neuromodulatory functions are mediated by the same cell in the trout. Our results indicate that widespread oxytocinergic innervation throughout the brain is a feature of other teleosts as well.

In the plainfin midshipman, oxytocin innervates the ventral Tel, and such fibers terminate in the olfactory bulb (Goodson et al., 2003). This olfactory innervation was not found in larval zebrafish here, but could appear in juvenile or adult animals. Similar to the trout, projections into the Tel also were largely restricted to its ventral part. It should be noted that putative amygdala-homologous regions are located more dorsally in the Tel (Wullimann and Mueller, 2004; Maximino et al., 2013) and therefore are probably not innervated by Oxt cells in larval zebrafish at this stage, but such innervation could develop later. The hypothalamic and tectal innervation described in the trout was also observed here in larval zebrafish. However, the projection pattern groups we observed in larval zebrafish are different from those in the trout, where oxytocinergic neurons innervate the pituitary and other brain regions simultaneously (Saito et al., 2004). The zebrafish oxytocin system could thus be more differentiated than its trout homolog. A separation similar to our results, however, was suggested for the plainfin midshipman (Goodson et al., 2003).

A second magnocellular cell type innervating the neurohypophysis in mammals produces the closely related neuropeptide Avp. We performed morphological analysis of vasopressinergic cells using the same approach. Our data suggest that vasopressinergic projections are less abundant in extrahypothalamic regions than oxytocinergic projections (Herget, Salazar Thula, and Ryu, unpublished data), as was also reported for descending projections in rats (Buijs, 1978; Sawchenko and Swanson, 1982) and in various fishes (Van den Dungen et al., 1982; Goodson et al., 2003; Saito et al., 2004). This suggests that Oxt might regulate more diverse brain functions than Avp in larval zebrafish. This difference in complexity is thought to be greater in evolutionarily more advanced vertebrates, in which magnocellular Avp neurons remained structurally simpler, whereas Oxt neurons acquired a more complex shape (Grinevich et al., 2016). However, there are numerous extrahypothalamic structures expressing Avp in rodents (BNST, medial amygdala, etc.), which can contribute to modulation of various behaviors (Rood and De Vries, 2011). In contrast, Avp somata in larval zebrafish are restricted to the NPO (Herget et al., 2014).

We previously found that a minor population of oxytocinergic cells coexpresses *crh* (rarely), *proenkephalin a* (rarely), or *proenkephalin b* (moderately) in 5 dpf larvae (Herget and Ryu, 2015). Consistent with our current results, cells displaying this peptide coexpression are localized to the rostral half of the Oxt cluster, which according to our reconstruction and registration approach features predominantly neuroendocrine cells projecting to the pituitary. These results suggest that the Oxt population consists of a rostral neuroendocrine group that partially coreleases enkephalins (and rarely Crh) into the pituitary and a caudal neuromodulatory group that innervates distinct brain regions to regulate behavior. This apparent spatial separation is not without precedent. Oxytocinergic axons of some cells in the caudal PVN in rats display collateral branches, in contrast to the predominantly hypophysiotropic cells of the rostral and lateral PVN (Sofroniew, 1983). Oxt neurons projecting to regions other than the pituitary also cluster in the dorsoposterior part of the PVN in prairie voles, and such neurons were suggested to innervate the brainstem, whereas neuroendocrine cells tend to localize in the anterior PVN (Ross et al., 2009). Therefore, the clustering of Oxt neurons in zebrafish may have a similar trend as reported in rats and voles, although the mammalian Oxt system is far more complex and includes numerous accessory nuclei that may have extrahypothalamic projections as well (Steinman et al., 2016). Oxytocinergic innervation of the Ce was previously demonstrated in the trout (Saito et al., 2004). In 6-dpf larval zebrafish, Oxt immunostaining showed considerable interindividual variability in the degree of innervation of various regions, and the Ce was not innervated in all animals (Fig. 3), but such innervation could still develop later in those animals.

The Oxt system has received considerable attention for many years due to its key role in social behavior. Oxt is thought to increase the salience of sensory information linked to social cues (Grinevich et al., 2016). This involvement of the Oxt system in socially relevant brain regions has been suggested for different sensory modalities in different animal groups (vocal regions in songbirds, olfactory regions in rodents, and visual regions in primates), further supporting the concept of a modulatory function of locally released oxytocin in regions important for the social behavior of that species (Grinevich et al., 2016). In sonic fish, which use vocal-acoustic systems for mating communication, the brain regions involved are innervated by oxytocinergic cells (Goodson et al., 2003). Also, in adult zebrafish, Oxt and Avp injections modulate social and anxiety-related behavior (Braida et al., 2012). However, potential roles of Oxt in larval zebrafish in the context of social behavior have not been explored so far. It is tempting to speculate that the oxytocinergic system is involved in the modulation of visually guided social behavior in zebrafish, given its projections into the TeO. Such fibers tend to be situated within the deep neuropil, adjacent to the stratum periventriculare. Therefore, the innervated layers are probably the stratum album centrale or stratum griseum centrale (Nevin et al., 2008; Scott and Baier, 2009; Baier, 2013). In the trout, oxytocinergic fibers are found in deep tectal regions along the stratum periventriculare as well (Saito et al., 2004). Oxytocinergic fibers are also found within the stratum album centrale and stratum griseum centrale in the midshipman (Goodson et al., 2003). Tectal processing studies suggest that the deeper neuropil layers primarily have an output function, receiving input from the superficial layers (Nevin et al., 2010). Deep tectal layers are thought to not only process visual input, but also receive extraretinal afferents from other brain regions and from different sensory systems (Kinoshita and Ito, 2006). Local modulation of visual processing in deep layers of the tectal neuropil by afferents from nonvisual brain regions was also suggested for zebrafish (Robles et al., 2011). Our results suggest that oxytocinergic cells of the NPO constitute one of these regions. The tectal innervation found here in larval zebrafish is in agreement with the notion that these animals rely primarily on visual cues for social behavior.

Further studies of the Oxt system in different stages and under different environmental conditions, such as social enrichment or social deprivation, could illuminate the potential for plasticity of Oxt circuits for social behavior. Further investigation and manipulation of oxytocinergic cells in zebrafish will greatly enrich our understanding of one of the most remarkable and relevant vertebrate peptide systems, potentially in a faster and more comprehensive way than is currently possible in rodents.

## Synthesis

Reviewing Editor: Jeffrey Blaustein, University of Massachusetts Decisions are customarily a result of the Reviewing Editor and the peer reviewers coming together and discussing their recommendations until a consensus is reached. When revisions are invited, a fact-based synthesis statement explaining their decision and outlining what is needed to prepare a revision will be listed below. The following reviewer(s) agreed to reveal their identity: Geert De Vries.

The reviewers and I agree that this is an interesting descriptive paper. Although it would be stronger with functional data as well, we believe there is value in the anatomical work. However, there are a number of issues that should be addressed as delineated in the reviewers comments.

Reviewer 1:

This work presents an anatomical characterization of the oxytocin system in zebrafish.

The authors examined general features of the distribution of fibers by immunostaining for oxytocin combined with NeuroTrace Fluorescent Nissl Stain for the identification of anatomical landmarks. In addition, they compared the general distribution pattern of oxytocin fibers between 8 individual fish (at 6 day old larvae), and found that in this stage of development there are some differences in the innervation of the cerebelum and tectum.

Next, the authors preformed a single cell genetic labeling with membrane-bond fluorophores. To this end they injected one-cell embryos with pST_Oxt:BrbM and hsp:nlsCre and induced mosaic recombination. Embryos were stained with a oxytocin antobodies and double positive cells were used for 3D reconstruction of the projecting neurons. Using this method the authors showed that the oxytocin neuronal system is highly heterogeneous in its innervation patterns. They classified the cells into two groups: pituitary-projecting cells (i.e. hypophysiotropic cells) and cells that project to other regions in the brain (i.e. encephalotropic cells).

OXT neurons regulate a wide range of processes including social interactions, stress, feeding, and water balance. These functions are conserved in vertebrates so characterizing the anatomy of this system and identifying the brain regions that it targets may contribute to our understanding of the manner by which it executes its function.

The work is of a descriptive nature and by and large the experiments were performed carefully and the image presentation is very esthetic. I have no major comment about presented data.

Minor remarks:

Figure 4 : the relevance/contribution of the Otp BrbM to the OXT mapping is not clear. As mentioned in the discussion, mapping of the OXT system was done based on a mosaic labeling of OXT cells in injected fish without exploiting the multi-color labeling of this system. In this context, Otp BrbM is even more unnecessary to this paper.

Figures 5+6: The authors present a registration of the pituitary projecting cells from different embryos. It is possible that there is a repeated labeling of the same cells thus a bias towards the labeling of certain types of cells (which leads to over/under representation of cells). Figure 6H which shows a combined registration of the pituitary labeled cells may not reflect the organization of these cells in embryos. Authors should refer to this in text/discussion. This remark also applies to figures 7+8.

Clarification question: The reconstruction is not symmetric. Is there laterality in the system?

Reviewer 2:

This is an elegant study of the oxytocin innervation of the zebrafish. Using the brainbow method, it shows in unprecedented detail the anatomy of hypothalamic projections toward pituitary and extrahypothalamic brain areas. It thereby forms a strong starting point to study the function of these pathways in an animal that offers a strong potential to manipulate these pathways genetically. As the authors don't seem to have a strong grasp on earlier literature, they overstate the importance of their findings and claim several 'firsts' that have been known for decades (see below). I agree with their use of the name 'oxytocin,' instead of 'isotocin,' which is typica used in older literature. As 'isotocin' is still commonly used, it warrants pointing out their choice in the introduction.

Specific comments:

47-49: Various groups have studied projections of magno- versus parvocellular cells in PVN (e.g., Swanson and Sawchenko in the 80s), with the former being involved in neuroendocrine functions and the latter being involved in central regulation of autonomic function. The statement that this 'is currently unknown' is therefore incorrect.

53-55: Their observations are therefore not as "remarkably" as they make it out to be.

55-57: "Complex target regions of oxytocin" were also known previously in fish. See, e.g., studies in midshipman fish by Goodson (2003) and in the rainbow trout by Van den Dungen (1982).

87-89: It has been known for over decades that oxytocin neurons have widespread projections to distinct brain areas and the spinal cord. Nothing of that literature is reviewed here. The reading of the literature seems to have been largely confined to research from the Grinevich lab and a rather restricted study in voles.

90-91: The hypothesis of dendritic release still stands. This hypothesis followed the earlier hypothesis that central oxytocin actions were mediated by extrahypothamic projections.

The unchallenged thought that mechanisms together account for central oxytocin action still stands. It would be interesting to use the genetic power of zebrafish to test this hypothesis.

104-106: The "currently open question is whether distinct Oxt neuron subtypes exist that mediate central vs. peripheral functions" has been answered long ago. (see e.g., Swanson Sawchenko papers). What is true, of course, is that this question is harder to tackle with the classic methods used, e.g., by Swanson and Sawchenko, who had the luxury of a relatively large brain. Similar studies are nearly impossible in fish. The results of the Brainbow methods are therefore beautiful, but not revolutionary, as suggested in line 317.

111-115 presents a more modest, but still very compelling, description of the specific contributions of this paper

314-317. (see comments to 47-49 above). By skipping the older literature, the authors miss an opportunity to related their findings with earlier ones regarding the location of neuroendocrine versus central projecting oxytocin neurons.

368-371: The statement that vasopressin projections are less complex than oxytocin projections goes completely against the literature, which has reported many more cell groups producing vasopressin than oxytocin (e.g., suprachiasmatic nucleus, bed nucleus of the stria terminalis, amygdala, and olfactory bulb) and projections to many more areas (See e.g., Buijs, De Vries, Rood, and in Fish Goodson and Van den Dungen).
